# A Multi‐Method Study to Develop and Pilot Test an Interprofessional Transitional Care Model for Frail Older Adults – AdvantAGE

**DOI:** 10.1111/jan.16822

**Published:** 2025-03-12

**Authors:** Isabel Pfundstein, Oliver Mauthner, Cynthia O. Gschwind, Olga Muser, Christian H. Nickel, Diana Trutschel, Thekla Brunkert

**Affiliations:** ^1^ University Department of Geriatric Medicine FELIX PLATTER Basel Switzerland; ^2^ Nursing Science (INS), Department Public Health (DPH), Faculty of Medicine University of Basel Basel Switzerland; ^3^ Department of Emergency Medicine University Hospital Basel Basel Switzerland; ^4^ Faculty of Health Sciences and Medicine University of Lucerne Lucerne Switzerland

**Keywords:** advanced practice nurses, implementation science, intervention development, logic model, transitional care

## Abstract

**Aim(s):**

To develop and pilot test the AdvantAGE transitional care model at a Swiss geriatric hospital.

**Design:**

Multi‐method design.

**Methods:**

The study progressed in three stages from January 2021 to December 2023: (1) contextual analysis using the Consolidated Framework for Implementation Research, incorporating qualitative interviews, (2) development and pilot testing of transitional care interventions on three acute geriatric wards using a descriptive explorative study design and (3) development and validation of a logic model using an iterative approach involving project interest groups and researchers.

**Results:**

We identified central challenges and needs related to transitions from hospital to home, including insufficient information flow, patient and caregiver insecurities and lacking adherence to recommended treatment. The newly developed transitional care model comprised five core elements: continuous support for patients and caregivers, care coordination with primary care providers, comprehensive health management at home, medication‐ and self‐management with patients and caregivers and advance care planning. Of 137 eligible patients, 62 participated in the 10‐month pilot test of the preliminary transitional care intervention, with an average participation duration of 69 days. Findings from the pilot informed the refinement of the intervention elements and the development of a preliminary logic model.

**Conclusion:**

Employing an implementation science approach facilitated the development and refinement of the AdvantAGE model, ensuring alignment with the needs of project interest groups and the specific implementation context.

**Impact:**

This study demonstrates the development of a transitional care model tailored to the specific needs and circumstances of the local healthcare context. Findings provide valuable insights for healthcare practitioners, researchers and policymakers, offering implications for developing transitional care practices and policies.

**Patient or Public Contribution:**

Limited patient and public involvement was incorporated, focusing on the interpretation of the findings of the first step of this study. Further contributions included providing feedback on the development of the elements of the AdvantAGE transitional care model, ensuring the research addressed priorities relevant to patients and primary health care providers in Basel‐Stadt.

**Summary**

- What already is known
  ○ Transitional care models (TCMs) aim to reduce rehospitalisations for frail older adults by enhancing care coordination and continuity.
  ○ Implementation challenges, such as misaligned care protocols or insufficient adaptation to local contexts, can hinder the effectiveness of TCMs.
- What this paper adds
  ○ A comprehensive process for developing a context‐tailored transitional care model using implementation science principles.
  ○ A description of practical challenges encountered during the pilot testing and recommendations for adaption.
- Implications for practice
  ○ The study provides a scalable, replicable framework for implementing TCMs that address local healthcare needs.
  ○ The logic model developed offers a structured tool for future evaluations, enabling the refinement and optimisation of TCM components.

## 1 Introduction

Frail older adults often face numerous health challenges that necessitate frequent medical consultations and lead to repeated hospitalisations. Transitional care models (TCMs) are designed to enhance coordination and continuity of care as patients move from hospital to community settings. By integrating strategies such as patient education, care coordination, and caregiver involvement, TCMs strive to improve patient outcomes, reduce rehospitalisations, and streamline the transition process. However, the effectiveness of these models is often hampered by implementation challenges. Studies suggest that modifications to TCMs, including the roles of healthcare providers, target groups and follow‐up care protocols, are frequently necessary to align with specific local needs. Implementation science offers a framework to address these issues by ensuring that interventions and implementation processes are adapted thoughtfully and based on a solid understanding of underlying program theory. The AdvantAGE project (Development and Implementation of an Advanced Practice Nurse‐led Interprofessional Transitional Care Model for Frail Geriatric Adults) aims to develop and implement a transitional care intervention aimed at reducing rehospitalisations by leveraging implementation science principles. This paper details the process of developing the AdvantAGE model; exploring the needs of older adults, caregivers and healthcare providers; pilot testing the model in a real‐world setting and developing a logic model. Through this approach, the project seeks to provide a scalable and replicable model for transitional care that is sensitive to the unique challenges of the local healthcare context.

## 2 Background

Acute care transitions from hospital to home present a challenge for frail older adults, as they are particularly vulnerable to rehospitalisation in the period after hospital discharge (Lilleheie et al. 2020). Addressing the complex needs of multimorbid and frail older patients necessitates integrated care approaches that ensure comprehensive and continuous care (Briggs et al. 2018). Central to this endeavour are TCMs, which serve as the cornerstone for enhancing coordination and care continuity as patients move from hospital to community settings. These models are designed to bridge care gaps and improve the outcomes of patients at risk, effectively smoothing the journey from acute care to home‐based recovery (Coleman and Boult 2003). TCMs are complex interventions that deploy a team‐based strategy to manage acute care transitions efficiently, ensuring a seamless patient journey (Naylor et al. 2018). Core elements of TCMs comprise among others: screening of at‐risk older adults, patient education, promotion of self‐management, care coordination, symptom and risk management and engagement of patients and caregivers (Naylor et al. 2018). A current review of integrated components of transitional care interventions highlighted that care models including ‘intensive follow‐up care, informal caregiver involvement, shared decision making and a small care team with a defined care coordinator’ were associated with more successful transitional care for frail older patients (Leithaus et al. 2022, 12). The growing body of research on TCMs for older adults demonstrates their effectiveness across various dimensions: At the system level, TCMs proved effective in reducing rehospitalisation rates and emergency admissions (Weeks et al. 2018), shortening hospital stays and decreasing mortality rates (Kutz et al. 2019). At the patient level, studies report enhancements in quality of life, and social and physical functioning (Zou et al. 2022). However, the heterogeneity of these studies makes direct comparisons of different TCMs and their specific elements challenging. A critical factor influencing effectiveness is the implementation process, which often faces hurdles such as poorly defined target populations (McGilton et al. 2021). A recent review by Fakha et al. (2021) underscores the importance of adapting implementation processes to meet contextual needs, summarising the main barriers and facilitators that impact the success of transitional care interventions.

Implementation science offers a methodological approach to effectively bridge the gap between research evidence and practical application in the real world by incorporating contextual factors into the development and implementation of interventions (Peters et al. 2013). Various frameworks within implementation science provide guidance on how to conceptualise contextual factors that potentially affect implementation (Nilsen and Bernhardsson 2019). A determinant framework, such as the updated Consolidated Framework for Implementation Research (CFIR) (Damschroder et al. 2022), can guide a thorough contextual analysis which is essential to inform the choice of implementation strategies that support the practical adoption of interventions (Powell et al. 2017). The CFIR comprises a list of 67 constructs that potentially influence implementation, which can be grouped into five domains: innovation, outer setting, inner setting, individuals and the implementation process (Damschroder et al. 2022). Furthermore, this preparatory exploration helps identify potential barriers, facilitators and needs, laying the groundwork for developing and adapting interventions that are contextually relevant. One central principle of implementation science distinguishing it from traditional clinical research is its focus on external validity. A well‐developed program theory, articulated through a logic model, lays a strong foundation for replicating and sustaining interventions across different contexts and scales (W. K. Kellogg Foundation 2004). The logic model maps out the expected causal pathways from intervention activities to outcomes, serving as a blueprint for monitoring and evaluation. This approach is instrumental in pinpointing key success indicators at different stages of the implementation process, enabling timely adjustments and informed decision‐making. Moreover, the development of a logic model enhances the engagement of project interest groups, another central principle of implementation science, by providing a visual and intuitive representation of how an intervention is intended to function, facilitating clearer understanding and support (Skivington et al. 2021).

In Switzerland, the population is steadily growing older, and individuals aged 65 and older represent the group most frequently re‐hospitalised, with a rehospitalisation rate of up to 10.2% (Havranek 2024). The Swiss governmental initiative, ‘Health 2030’, promotes integrated care as a solution to address upcoming healthcare challenges. Currently, however, there is a notable gap in transitional care for frail older adults, with no existing models extending care from the hospital to the patient's home. The AdvantAGE project aims to address this gap by developing, implementing, and evaluating a TCM employing an implementation science approach to ensure effective translation of evidence to the Swiss healthcare system. This study reports on the first phase of a larger collaborative project between the cantonal health department and a Swiss geriatric hospital.

### 2.1 Aims and Objectives

This study aims to develop an interprofessional transitional care model at a Swiss geriatric hospital with the goal of reducing rehospitalisation rates in frail older adults transitioning from hospital to home. The specific aims of this study are (1) to explore challenges and needs of older adults, their informal caregivers and healthcare providers regarding the transition from hospital to home (contextual analysis); (2) to develop and pilot test intervention elements and (3) to develop a logic model of the contextually adapted transitional care intervention.

## 3 Method

### 3.1 Design and Methodology

**FIGURE 1:**
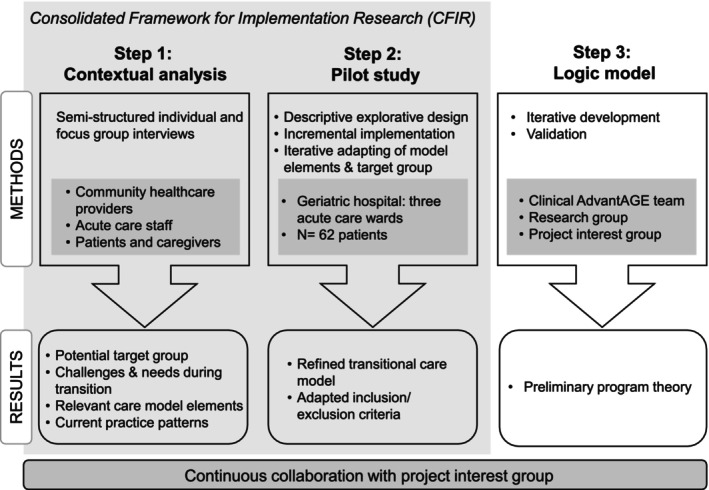
Overview of the three‐step process to develop and pilot test the new transitional care model, and to develop a logic model.

This study uses a three‐step process combining qualitative and quantitative data to inform the development of a transitional care model following the Medical Research Council (MRC) framework (Skivington et al. 2021). In the first step, we conducted a contextual analysis, collecting and analysing qualitative data. Based on these findings, we developed preliminary intervention elements of the TCM and determined eligibility criteria. In the second step, we pilot tested these intervention elements using a descriptive explorative design. In the third step, we iteratively developed a logic model during the period of pilot testing. Figure 1 provides an overview of this study's approach.

### 3.2 Ethical Considerations

We received approval for conducting the contextual analysis (Step 1) of this study by the ethics committee of Northwestern and Central Switzerland (EKNZ‐2021‐01471). Further, this ethics committee issued a declaration of no objection to the pilot test (Step 2) as the latter is not defined as a research project with humans as per Human Research Act, art. 2 (‘Human Research Act, HRA’ 2011).

### 3.3 Study Setting

The AdvantAGE project is implemented within the University Department of Geriatric Medicine FELIX PLATTER (UAFP) in the Canton Basel‐Stadt, Switzerland. The UAFP provides inpatient and outpatient services across acute geriatric medicine, geriatric psychiatry and rehabilitation. Medical care is delivered by interprofessional teams adhering to a bio‐psychosocial approach. Annually, the UAFP treats approximately 5000 inpatient cases and serves over 3000 outpatient visits. As part of standard practice, all new inpatients at UAFP undergo a comprehensive geriatric assessment that helps in crafting individualised care and treatment plans, including coordinated discharge planning. A preliminary analysis of UAFP inpatient data from 2019 to 2021 showed an average rehospitalisation rate of 22%. Currently, there is no specific focus on transitional care or support post‐discharge. To address this gap, in early 2022, UAFP and the cantonal health department of Basel‐Stadt reached an agreement to initiate transitional care services tailored for community‐dwelling older adults post hospitalisation. This service, funded by the Canton of Basel‐Stadt for a period of 36 months, involves a dedicated transitional care team comprising four Advanced Practice Nurses (APNs) with full‐time equivalents (FTE) of three, a social worker (FTE 0.5), a senior physician/geriatrician (FTE 0.2), a nutritionist (FTE 0.1) and an occupational therapist (FTE 0.1).

### 3.4 Step 1: Contextual Analysis

Our contextual analysis was conducted using the Basel Approach for Contextual Analysis (BANANA) as outlined by Mielke et al. (2022). This comprehensive approach consists of six distinct steps: (I) selecting a theory, model or framework to guide the analysis; (II) reviewing empirical evidence on relevant contextual factors, including facilitators, barriers and practice patterns that affect both implementation and the intervention itself; (III) engaging relevant stakeholders; (IV) collecting and analysing data; (V) identifying and elucidating the relevance of contextual factors for the co‐design of interventions, implementation strategies and outcomes and (VI) formally reporting the findings of the contextual analysis. In our study, we utilised the Consolidated Framework for Implementation Research (CFIR) to guide the overall project (step I). To inform data collection and discussions with project interest groups, we conducted a review of current evidence on relevant contextual factors (step II). Due to space constraints, this manuscript will specifically focus on our engagement with project interest groups (step III) and the collection and analysis of qualitative data (steps IV, V and VI).

#### 3.4.1 Formation of a Project Interest Group/Engaging With Project Interest Groups

To ensure the fit of the new care model with the local context and alignment with the needs of all interest groups, a project interest group was formed in early 2021. The group includes representatives from four distinct sectors: (1) the hospital setting, which includes geriatricians, nurses, social workers, therapists and hospital management; (2) the primary care setting, which encompasses general practitioners (GPs), pharmacists, home care and social care organisations; (3) the cantonal health department and (4) representatives of older people and caregivers. The group meets on a regular basis (every 4–6 months) to discuss emerging study findings and their implications for the ongoing project, considering the local context.

The initial meeting with the project interest group was aimed at introducing the project's background and familiarising ourselves with the group members. At our second meeting, we facilitated small group discussions to delve into their specific needs concerning transitional care in the local context and to refine our understanding of the potential target group. These discussions were initiated with a brief presentation of the preliminary results from our qualitative data collection. To foster interactive engagement, we invited the project interest group to contribute their suggestions on posterboards. One set of posterboards displayed a list of elements from transitional care programs for older adults, as outlined by McGilton et al. (2021). Another set summarised potential inclusion and exclusion criteria derived from the literature, which were further enriched by insights from our qualitative data.

As the project progressed, we continually shared summaries of our preliminary findings with the project interest groups, inviting their feedback and addressing any concerns they might have regarding the implementation of the new care model. In addition to the regular group meetings, we also held bilateral conversations addressing specific concerns. This iterative feedback process was essential for refining our approach and ensuring the relevance and feasibility of the developed care model.

#### 3.4.2 Qualitative Data Collection

The qualitative component of the contextual analysis incorporated a blend of individual and focus group interviews, targeting three distinct cohorts: patients and/or their informal caregivers (A), acute care staff (B) and community healthcare providers (C). To facilitate these discussions, we crafted semi‐structured interview guides, each uniquely tailored to capture the respective group's insights on the transitional care phase post‐hospitalisation. Central questions guiding the interviews were: What patient group experiences frequent rehospitalisations and could benefit the most from receiving transitional care? What are the frequent challenges encountered during the transition from hospital to home and how could these be addressed in a transitional care model?

#### 3.4.3 Sampling Strategy

##### 3.4.3.1 Patients and Informal Caregivers (A)

We employed a maximum variation sampling strategy to ensure a wide range of patient perspectives, particularly those potentially eligible for a new transitional care model. This approach allowed us to include participants with diverse living situations and support levels, including individuals living alone, with partners, or those receiving varied levels of informal care. Eligibility criteria centred on patients from acute or rehabilitation wards who reside in Canton Basel‐Stadt, with exclusions for cognitive impairment [Mini–Mental State Examination score (MMSE) < 21] (Folstein et al. 1975) or terminal illnesses. In cases where the patient met the criteria for inclusion but had a cognitive impairment, only the informal caregivers were considered for an interview.

##### 3.4.3.2 Acute Care Staff (B)

A purposive sampling method was utilised to recruit healthcare staff from acute and rehabilitation wards, focusing on those with direct patient contact and at least 6 months of experience at the hospital. This strategy aimed to capture a broad spectrum of professional roles and insights relevant to the transitional care model's future implementation.

##### 3.4.3.3 Community Healthcare Providers (C)

To identify community healthcare providers, we adopted a convenience sampling approach, engaging with representatives from community pharmacies, home care organisations and general practitioners.

This structured approach to qualitative data collection is designed to yield comprehensive insights into the experiences and needs of all local healthcare partners involved in the transitional care process, thereby informing the development and implementation of effective transitional care models.

#### 3.4.4 Participants and Data Collection Methods

##### 3.4.4.1 Patients and Informal Caregivers (A)

We engaged with patients and their caregivers through a series of three interviews to capture in‐depth feedback on their transitional care experience. The initial interview, conducted prior to discharge, explored their anticipations and concerns regarding the upcoming transition. Subsequent interviews were held at the participants' homes 1‐ and 4‐weeks post‐discharge, focusing on the actual transition experience, challenges encountered, and unmet needs. Each interview, ranging from 30 to 60 min, was led by a research assistant with a master's degree in physiotherapy and foundational training in qualitative research methods.

##### 3.4.4.2 Acute Care Staff (B)

Within the hospital setting, we organised focus groups with nurses and therapists, including a diverse range of positions and specialties. These sessions and all other interviews were facilitated by a postdoctoral researcher experienced in qualitative methodologies, employing a knowledge mapping approach to structure discussions and capture insights (Pelz et al. 2004). On average, the focus groups lasted 55 min. Furthermore, we conducted individual interviews with senior physicians lasting 35–60 min.

##### 3.4.4.3 Community Healthcare Providers (C)

Individual interviews with community healthcare providers (e.g., pharmacists, home care organisation representatives, general practitioners) were conducted either in person or via telephone, depending on the participants' preference. These interviews lasted between 40 and 60 min.

All interviews across groups (A, B and C) were audio‐recorded, with field notes taken to complement the recordings.

#### 3.4.5 Qualitative Data Analysis

The analysis of our interviews utilised Rapid Assessment Procedures (RAP) within a collaborative team‐based approach as described by Vindrola‐Padros (2021) and Rankl et al. (2021). Following each interview, the respective interviewer and a research assistant populated a spreadsheet with information categorised according to predefined criteria. Audio recordings were transcribed into a RAP sheet, and content was added by a second researcher. From the beginning until the completion of data collection, an iterative process was used to discuss the interview content within the research team. In the first step, a preliminary content analysis by the individual research team members took place to identify the initial codes, which were further elaborated in a discussion among the research team. The further elaboration of the codes and themes was regularly discussed in the research team and modified if necessary.

Socio‐demographic data, for example, sex, age, years of work experience, etc., of focus group and individual interview participants were assessed (Table S1a,b).

### 3.5 Step 2: Pilot Study

#### 3.5.1 Design

We adopted a descriptive exploratory study design.

#### 3.5.2 Aims

The primary aim of this pilot study was to identify and address challenges related to the intervention and its implementation. Specific objectives included assessing recruitment rates, evaluating the appropriateness of inclusion and exclusion criteria, determining the feasibility of the intervention elements and identifying potential adaptations needed for the implementation processes.

#### 3.5.3 Setting

**FIGURE 2:**
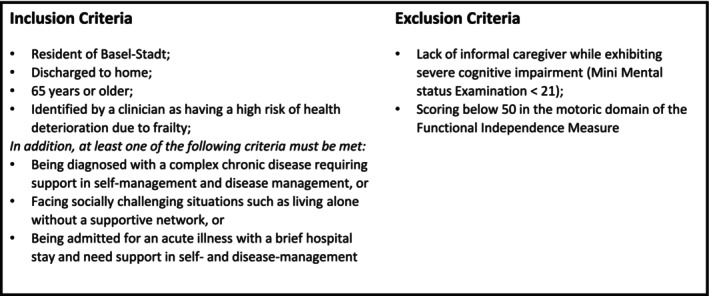
Inclusion and exclusion criteria based on findings from step 1.

The pilot study was conducted within three acute geriatric wards of the UAFP. Initiated at the end of February 2023, the study ran for a duration of 10 months, concluding at the end of December 2023. Initially, the intervention was introduced on a single ward containing 43 beds. After 2 months, the pilot was expanded to include an additional ward with 39 beds and, following 8 months, expanded further to incorporate a larger ward with 56 beds. Patients were screened according to the preliminary eligibility criteria (Figure 2) which were derived from the findings from the preceding context analysis (Step 1). This phased expansion allowed for gradual integration of the care model and iterative refinement based on early outcomes and feedback.

#### 3.5.4 Intervention

The newly developed transitional care model is designed to reduce the risk of unplanned readmissions. It incorporates five core elements based on initial findings from Step 1: (1) continuous support for patients and caregivers, (2) care coordination with primary care providers, (3) comprehensive health management at home, (4) medication‐ and self‐management with patients and caregivers and (5) advance care planning. These elements are primarily delivered through home visits by the APNs, who lead the transition process for each participant. The intervention lasts up to 90 days but can conclude earlier if the patient's transitional care needs are fully addressed. The social worker facilitates access to financial and social support, while nutritional and occupational therapists provide tailored consultations to address specific dietary and environmental needs. Physicians contribute geriatric expertise, focusing on symptom and medication management and providing supervision for the APNs. Furthermore, the interprofessional AdvantAGE team regularly convenes to discuss complex cases and develop individual solutions for patient's challenges.

#### 3.5.5 Implementation Strategies

We adopted a range of different implementation strategies to cater for the information and support needs of the immediate project context identified in step 1 of our project. The strategies were selected from eight different ERIC (Expert Recommendations for Implementing Change) clusters (Waltz et al. 2015): (1) Use evaluative and iterative strategies, (2) adapt and tailor to context, (3) develop stakeholder interrelationships, (4) train and educate stakeholders, (5) change infrastructure, (6) provide interactive assistance, (7) support clinicians and (8) engage consumers. We promoted acceptance of the new TCM through regular engagement with the project interest groups and local coalition building, such as meetings with the local home care organisation and the AdvantAGE team. In addition, we conducted several coaching and training sessions for the clinical AdvantAGE team aiming at preparation for the new roles, and the APNs received training focusing on geriatric symptoms and clinical assessment skills to equip them with the required skills to conduct all intervention elements. Furthermore, we gradually expanded the new TCM in the hospital to address emerging challenges and to adapt and refine processes as needed. At the same time, we were raising awareness of the AdvantAGE project among UAFP staff through verbal briefings by the clinical AdvantAGE team, complemented by written information provided to target wards. Since the start of the pilot phase, we surveyed patient satisfaction with the intervention to ensure quality and timely adaptations if necessary.

#### 3.5.6 Data Collection and Analysis

Throughout the pilot phase, we collected a range of data from different sources comprising primary data gathered by the project APNs and routine data from the hospital's patient documentation system to obtain sociodemographic information of the participants, that is, age, sex and living situation at home.

##### 3.5.6.1 Individual Intervention Times

We defined and recorded the duration of the intervention as the number of days from the first home visit to the last for each participant based on the screening log. Furthermore, we assessed the number of home visits conducted by the APNs.

Rehospitalisation events: In cases of rehospitalisation, the admission date was recorded for each affected participant. Subsequently, a standardised root‐cause analysis (RCA) was conducted by the APN in charge of the readmitted patient. The root‐cause process involved completing a detailed document that included open‐ended questions and multiple‐choice options to assess the circumstances of the readmission, its preventability and areas for potential improvement. This document served as a basis for discussion among the multidisciplinary team to derive actionable insights.

We conducted the descriptive statistical analysis, calculating means and medians for continuous variables and proportions for categorical data to summarise and understand patterns within the collected data effectively.

### 3.6 Step 3: Development and Validation of the Logic Model

To guide the implementation and evaluation of our TCM, we developed a logic model that delineates the input, activities, anticipated outcomes and impact, articulating the overarching program theory. This model was designed to visually represent how our complex intervention is expected to achieve desired results. The framework for the logic model was adapted from established guidelines by the W. K. Kellogg Foundation (2004), which provides a structured approach to mapping out the components of a program: (A) Inputs: Resources necessary for program implementation, such as staffing, funding and infrastructure/materials; (B) Activities: Key actions or processes that the program undertakes to drive change (= intervention elements); (C) Outcomes: Classified into short‐term (level of processes/patients and caregivers), intermediate and distal, describing the direct results and effects of the intervention elements and (D) Impact: Ultimate goal or effect of the program on the community.

The development of the logic model employed a deductive approach, where theoretical and practical elements were synthesised from various sources including relevant literature, contextual analysis data, experiences from the pilot study and program documentation. Recognising that the development of a logic model is an iterative process, our model was continually refined as new contextual information was acquired, gaps in the program theory were identified, and additional intervention elements were recognised as necessary.

## 4 Results

### 4.1 Step 1: Contextual Analysis

In our study, we conducted a total of 43 interviews and three focus groups, including patients/caregivers, hospital staff and community healthcare providers to explore the transitional care experience comprehensively. Specifically, we carried out 18 longitudinal interviews with six patients and/or their caregivers, three focus groups and seven individual interviews within the hospital setting and 15 interviews with community healthcare providers. Detailed participant characteristics are outlined in Table S1a,b.

#### 4.1.1 Target Group for the New Care Model

The interviewees frequently identified patients with complex medical conditions as a key group that could benefit from transitional care. GPs and hospital physicians often mentioned heart failure and chronic obstructive pulmonary disease, noting that these conditions require high levels of self‐management to maintain stable health. Other conditions highlighted by healthcare providers included diabetes mellitus and epilepsy, where complex medication regimens necessitate significant self‐management. Patients with high care or support needs due to limited cognitive and functional capabilities were also suggested as a group with transitional care needs, as they are at an increased risk of rehospitalization.

Physicians emphasised that frail patients admitted for acute illnesses, such as pneumonia or fractures, often have prolonged care needs that require more intensive follow‐up. Despite being discharged in stable conditions after relatively short hospital stays, these patients are perceived to be at high risk of relapse or decline in functional capabilities.

Beyond medical conditions, healthcare professionals noted that patients living alone and without a supportive network face more difficult transitions, often resulting in rehospitalisations. The importance of caregivers and family support was repeatedly emphasised. Interviewees pointed out that not having caregivers, having overwhelmed caregivers, or having caregivers in disagreement about the care plan posed significant risks. Additionally, patients without a regular GP were identified as being at higher risk for rehospitalisation.

Several healthcare professionals, including GPs, nurses, social care workers and therapists, highlighted patients who refuse additional care services—such as home care, moving to a nursing facility or regular GP visits. These refusals were often based on the patients' perceptions that they did not require support, their tendency to trivialise their conditions or their desire to return home against medical advice. While a few participants suggested that this patient group might not benefit from transitional care, most believed that TCM could be especially helpful in rebuilding trust and ensuring these patients receive the necessary care.

#### 4.1.2 Challenges Encountered During the Transition From Hospital to Home and Potential Measures

The most frequently reported challenge related to transitions was insufficient information transfer between care settings and healthcare providers. This information gap primarily concerned medication details, recommended therapies, and post‐discharge follow‐ups. GPs frequently encountered changes in medication regimens made during hospital stays without adequate explanations in discharge letters, making it time‐consuming to determine the reasoning behind these changes. Patients also felt inadequately informed, often not knowing when to see their GP for follow‐up checks, how to organise recommended treatments or how to obtain prescribed medications. Some informal caregivers reported that they had to bridge the information gap between various healthcare providers, which resulted in them feeling highly burdened by this coordinating role.

Additionally, patients and caregivers frequently did not know whom to contact if symptoms worsened or if they needed health‐related advice, leading to feelings of helplessness and being overwhelmed. Interviewees from all groups pointed out that having one central contact person could address the issue of insufficient information flow. This individual should maintain regular communication with the patient and their caregivers, coordinate with other primary care providers and the hospital, build a trustworthy relationship and provide continuous support. Healthcare providers highlighted these concerns, stating that not enough support is offered to relieve caregivers and emphasising the need for additional assistance. They suggested better coordination among the different players involved in the patient's care, starting before discharge. This coordination should ensure that recommended therapies and follow‐ups will be carried out by facilitating communication among primary care providers and institutions.

Patients, caregivers and healthcare providers noted patients' frustration and insecurity regarding their declining health status, which affected their ability to perform daily activities. A few informal caregivers mentioned the need to make changes in response to deteriorating health, though they were unsure about what those changes would be. To help patients manage daily life post‐discharge, healthcare providers suggested to promote patient and caregiver's self‐efficacy in handling their chronic conditions as part of the new transitional care services. Potential interventions could include medication management training, checking prescriptions and intake and educating patients on self‐management. Further, it was discussed that this support should start in the hospital and continue at home, particularly for chronic diseases and medication management.

To address the need for ongoing support, the concept of post‐discharge follow‐up checks at home was highlighted by all interviewees. This could involve home visits, regular phone calls to monitor health status, assessing daily activities and living situations, monitoring symptoms such as weight checks for cardiac patients and communicating emerging needs to relevant healthcare providers. Caregivers and patients also expressed a strong need for a medical professional who would be present at home. While all interviewed GPs conducted home visits themselves, they highlighted their immense value but at the same time noted their time‐consuming nature. Thus, they would highly appreciate if another healthcare professional with sufficient medical knowledge could take over the home visits after hospital discharge and report any assessment results or concerns back to the GPs.

### 4.2 Step 2: Pilot Study

#### 4.2.1 Recruitment and Participation Time

**FIGURE 3:**
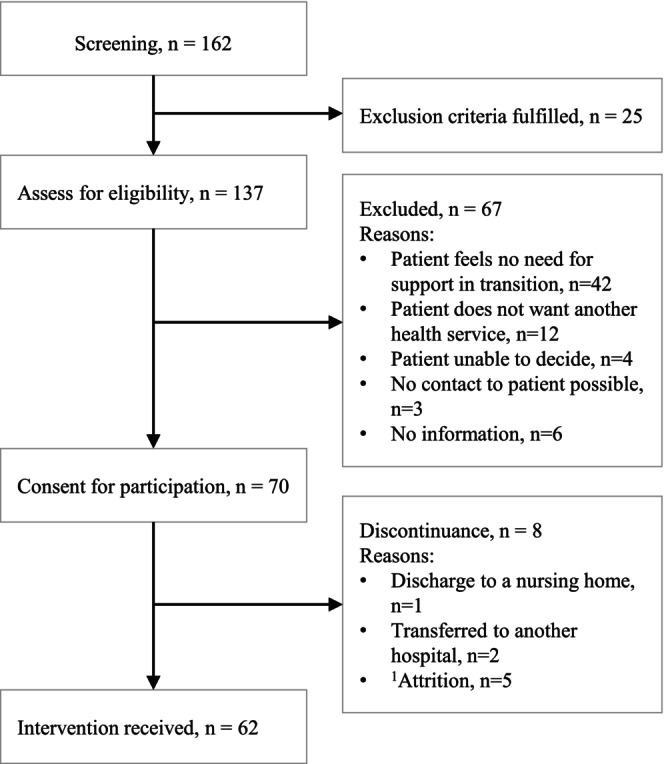
Flow diagram of study participants. *n* = number of patients. ^1^Attrition: Includes patients who consented during their hospital stay to participate in AdvantAGE but changed their opinion before receiving a home visit from the APN.

On the participating wards, healthcare staff identified 162 patients as potentially eligible for the new care model. Of these, 25 did not meet all the eligibility criteria. Among the 137 patients who did meet the criteria, 67 opted not to participate; the most frequently cited reason was a perceived lack of need for post‐discharge support, noted by 42 patients. Consequently, 70 patients were enrolled in the program, of whom 8 discontinued their participation before the first home visit. Ultimately, 62 participants received the AdvantAGE intervention. The demographics and clinical characteristics of these participants are detailed in Table 1. For further details about the recruitment, refer to Figure 3.

**TABLE 1:** Characteristics of patients of the pilot study.

	All included patients (*n* = 62)	Rehospitalised patients (*n* = 14)	Non‐rehospitalised patients (*n* = 47)
	*n* (%)	*n* (%)	*n* (%)
Demographics			
Female	38 (61.3%)	9 (64%)	29 (60.4%)
Age, years, mean (SD)	85 (6.95)	86 (8.33)	85 (6.49)
Living situation at home			
Living alone	54 (87.1%)	11 (79%)	43 (89.6%)
Living with partner	8 (12.9%)	3 (21%)	5 (10.4%)
Intervention			
Number of home visits, mean (SD)	10 (5.72)	8 (4.8)	11^b^ (6.13)^b^
Duration of intervention^a^, days, mean (SD)	69 (32.95)	56 (39.15)	75^b^ (30.55)^b^

Abbreviations: *n*, number of participants; SD, standard deviation.

^a^ Duration of intervention was calculated from the first home visit to the last home visit.

^b^ One patient was only cared for by telephone and had no home visits; therefore, it was omitted in the calculation of intervention duration.

The duration of each patient's participation in the AdvantAGE care model varied widely. On average, the intervention lasted 69 days per patient, with the number of home visits ranging from 1 to 29, highlighting the variability in patient needs across the study cohort.

#### 4.2.2 Rehospitalizations

During the 10‐month pilot phase, 14 out of the 62 participants that received the intervention experienced an unplanned readmission, representing 22.6% of the cohort. Of these readmissions, two patients were subsequently transferred directly to nursing facilities following their unplanned hospital stays. The average time between hospital discharge and unplanned readmission was 37 days (standard deviation ±23.01).

Following each readmission, the multiprofessional team conducted a root‐cause analysis (RCA) to explore the underlying reasons. Based on these RCAs, the team determined that four of the readmissions were avoidable. Common to these cases was a lack of knowledge on the part of patients and their caregivers about how to manage their conditions at home and uncertainty about whom to contact in emergent situations, leading to reliance on ambulance services. The remaining 10 readmissions were considered unavoidable, attributed to factors such as injuries from falls, progression of chronic diseases and acute infections (COVID‐19). Each RCA provided valuable insights, leading to ‘lessons learned’ that prompted various adaptations in the care model. These adaptations aimed to enhance the support system for patients and caregivers, thereby reducing the likelihood of future avoidable readmissions.

#### 4.2.3 Adaptations

The pilot phase provided valuable insights, leading to several important adaptations in the eligibility criteria and the intervention elements of the new AdvantAGE care model.

##### 4.2.3.1 Eligibility Criteria Adjustments

Based on recurrent experiences reported by the APNs, we introduced the criterion ‘having a psychiatric disorder that significantly impacts their ability to manage daily life’. This decision stemmed from observations that the AdvantAGE intervention was not adequately suited to meet the specialised needs of this patient group, resulting in minimal benefits.

The inclusion criterion ‘being able to speak and comprehend German’ was added. Effective communication in German is essential for participating in assessments and fully benefiting from the intervention's educational elements.

Furthermore, we raised the threshold for the MMSE from 21 to ≤ 23. This adjustment aligns with literature suggesting that the cognitive ability to consent to participate in a study is compromised at an MMSE score of 23 or lower (Felnhofer et al. 2013).

##### 4.2.3.2 Refinement of Intervention Elements

In the course of the pilot study, the AdvantAGE team encountered recurrent challenges related to the high level of patients' need for counselling, monitoring and coordination of care. Based on these challenges, we adapted the intervention by specifying and refining operationalisations considered minimal requirements for each core element of the TCM (detailed in Table S2). Table 2 outlines the major challenges that were identified during the pilot phase and the consequent adaptations to the intervention. One significant challenge was the long intervals between home visits, which led to changes in patients' health going unrecognised. To address this, we established a minimum frequency of contact within the first month after hospital discharge. Another critical issue identified during the pilot phase was medication discrepancies caused by inconsistencies in prescribing or taking medication, lack of information and other factors. While the APNs addressed the patient's medication during home visits, it became clear that a more structured approach was necessary to avoid unnecessary discontinuation of medication. Therefore, we introduced a medication review as an additional intervention element for the first home visit. During this review, APNs conduct a brown bag review (Larrat et al. 1990) at the patient's home, comparing available medication with the medication plan from the hospital and/or any updated medication list from the pharmacy or GP. Any discrepancies are immediately addressed by the APNs through conversations with the patient, their caregivers, the respective GPs or pharmacists.

**TABLE 2:** Challenges and adaptation to the AdvantAGE care model during the pilot phase.

Challenges	Adaptations to the intervention
Limited self‐efficacy in handling acute situations: – Patients do not know how to manage worsening symptoms – Patients cannot decide whether a health issue requires immediate medical attention – Patients are unsure whom to contact in case of acute health status change or worsening symptoms	– A document listing emergency contact numbers is provided to the patient during the first home visit – Patients are provided with information on individually relevant symptom management before discharge – Instruction on symptoms and symptom management is given by the second home visit – At least once during the AdvantAGE program, a detailed consultation is conducted focusing on managing one or more chronic conditions (including identifying symptoms and managing them) – The patient's current medication list, the hospital discharge letter, any existing advance directives and the GP's telephone number are placed in the patient's home where they are easily accessible to other caregivers in emergencies
Missed deterioration in health status of patients due to long intervals between home visits	– During the first 4 weeks after discharge, at least one home visit per week is planned
Discrepancies between medication prescriptions and patients’ actual medication intake	– During the first home visit: ○ The APN ensures the availability of a current medication list ○ The APN ensures the availability of the prescribed medications ○ Patients are asked to display all medications they have at home (brown bag review) – Any discrepancies between prescribed medications and actual intake are documented and addressed
Delayed implementation of hospital treatment recommendations	– All interventions recommended in the discharge letter are coordinated within 1 week of discharge

In addition to these adaptations, we also readjusted our implementation processes. Discussions with the clinical AdvantAGE team revealed that therapy suggestions or adaptations made in the hospital were not being promptly implemented after discharge. Examples include scheduling timely appointments with the GP and other primary care providers and coordinating additional care services or medical examinations. To address these issues, we added more explicit time intervals to the minimal requirements; on the other hand, we incorporated these aspects into coaching sessions with the APNs.

### 4.3 Step 3: Logic Model

**FIGURE 4:**
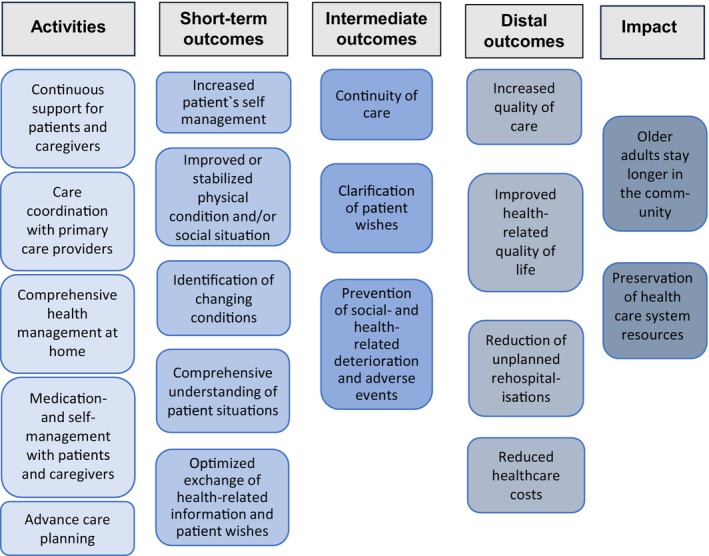
AdvantAGE program theory, summarised.

**FIGURE 5:**
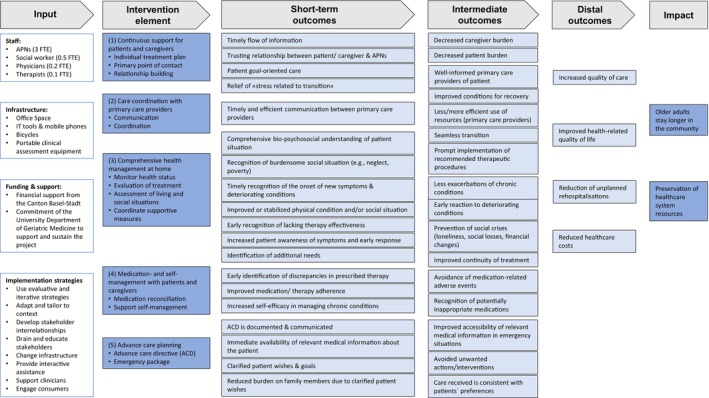
AdvantAGE program theory in detail.

The logic model illustrates our hypotheses regarding how each core element of the TCM contributes theoretically to achieving the model's objectives. Figure 4 offers a summarised visual representation of the programme theory, while a detailed illustration of all inputs, activities and outcomes is provided in Figure 5.

We propose that our core elements are instrumental in improving or stabilising the patient's physical and social conditions, as well as enhancing self‐management capabilities. This improvement might include increased symptom awareness, achieved through the educational and counselling components of the intervention. Short‐term outcomes also entail a thorough understanding of the patient's overall situation, including any burdensome social circumstances, early recognition of changes in condition such as ineffective therapies and optimised communication of health‐related information and patient preferences.

Over the intermediate term, we anticipate that various elements of the TCM will prevent the deterioration of social and health conditions and avert adverse events, such as medication‐related complications or worsening of chronic illnesses. Additionally, we expect these elements to clarify patient wishes and enhance continuity of care, for instance, through improved access to crucial medical information in emergencies. Ultimately, our goals include reducing rehospitalisation rates, improving the health‐related quality of life and quality of care for home‐dwelling older adults and decreasing healthcare costs. The anticipated impact of these outcomes is that older adults can stay in the community longer, thus alleviating the financial burden on the health care system and enhancing the efficiency of resource utilisation.

## 5 Discussion

The AdvantAGE project was initiated with the goal of developing a context‐specific transitional care model to lower rehospitalisation rates among frail older adults transitioning from hospital to home. This paper describes a three‐step process in the development of the TCM: Initially, we identified key challenges and needs related to transitions from hospital to home, such as interruptions in the information flow, feelings of burden and insecurity in patients and caregivers and the need for a single coordinating person. Subsequently, we pilot tested the newly developed transitional care intervention with 62 patients to assess the feasibility of the eligibility criteria and intervention elements. During the pilot phase, we made several adjustments in response to the challenges encountered, culminating in the creation of a logic model that links the TCM's intervention elements to its potential and desired outcomes.

One significant obstacle frequently reported in the implementation of TCMs is the absence of clear definitions for the target group (Fakha et al. 2021). One strategy to address this issue is employing risk assessment tools such as the HOSPITAL (Donzé et al. 2013) or LACE (van Walraven et al. 2010) scores, which identify patients at risk of rehospitalization. While these tools effectively highlight risks associated with pre‐existing medical conditions or previous admissions, they fall short in providing strategies to mitigate these risks. Moreover, their effectiveness has not been validated in a predominantly geriatric population. Therefore, relying solely on these scores to identify our target group for the AdvantAGE project would have been inadequate. Instead, we adopted a comprehensive approach that integrated insights from our context analysis, stakeholder engagement, and observations from the pilot phase. This approach allowed us to refine our eligibility criteria and tailor the intervention elements to suit the specific needs of our target population within the local context.

During the pilot study, we observed that a subset of participants, despite being identified by hospital staff as requiring substantial support from home care teams or institutional care, opted to receive the AdvantAGE intervention as their only form of support. This choice led to diverse outcomes: for some, the program acted as a gateway to engaging other healthcare services, while others remained reluctant to accept any changes to their care, preventing the delivery of certain intervention elements despite extensive efforts by the APNs. This variability in outcomes prompted the adoption of a case‐by‐case approach in inclusion decisions, made in collaboration with hospital staff and grounded in ethical considerations.

Simultaneously, the pilot study highlighted the critical importance of individual components in transitional care, as identified in existing research (Leithaus et al. 2022; McGilton et al. 2021). Notably, we found that establishing a trusting relationship between the APN and the patient was pivotal. Patients who had already formed a trusting relationship with an APN during their hospital stay were more receptive to the AdvantAGE program, even if they initially resisted other recommended health services. This finding contrasts with a recent review indicating frequent adaptations in TCMs that involve different healthcare providers visiting patients across settings (Naylor et al. 2018). However, our experience underscores the significance of maintaining continuity with a trusted healthcare provider, suggesting that this element should be preserved, as it can be essential for some patients to accept care.

Adaptation is a critical element in the implementation process, essential for increasing feasibility, acceptability and enhancing contextual fit (Damschroder et al. 2022). While our pilot study was designed to facilitate iterative adaptations, we anticipate the need for ongoing adjustments to meet emerging needs and contextual challenges. However, to conduct a robust evaluation, it is crucial to maintain consistency in the delivery of interventions. Monitoring fidelity within complex interventions poses challenges due to the multifaceted nature of these interventions, their interactive components and the necessity for variation tailored to individual patient needs (Skivington et al. 2021). To address this, our strategy involved the iterative development of minimal requirements for each intervention element, a key part of our comprehensive development approach (Table S2). An intervention typically comprises core functions, which are the central mechanisms of an intervention element and forms, which are the specific steps undertaken to execute these core functions (Perez Jolles et al. 2019). In this study, the minimal requirements are focused on the core functions, allowing us to monitor intervention fidelity while still accommodating necessary adaptations in the delivery method. This balanced approach ensures that our model remains both robust and flexible, providing a strong foundation for adapting the developed TCM to other contexts in the future.

### 5.1 Strengths and Limitations

This study presents several strengths and limitations that are critical to its evaluation and potential replication in other settings. It employs a multi‐methods approach that provides a comprehensive understanding of the needs and challenges faced by older adults, caregivers and healthcare providers. This ensures that the developed model is responsive and tailored to real‐world conditions. The phased approach allows for iterative refinements based on continuous feedback, enhancing the model's effectiveness. Extensive engagement of a project interest group is a major strength, as it incorporates the perspectives of all relevant parties, enhancing the model's relevance and acceptability. The study emphasises external validity, using the CFIR to consider various contextual factors that affect implementation (Damschroder et al. 2022). Additionally, the development of a logic model offers a clear visualisation of the intervention's expected pathways and outcomes, aiding in monitoring and evaluation and serving as a valuable tool for communicating the program theory to stakeholders and funders. However, the study also faces some limitations that must be acknowledged. While it provides valuable insights, the results are primarily applicable to the Swiss healthcare context, specifically within the geriatric population of Basel‐Stadt. This limited scope may affect the generalisability of the findings to other regions or healthcare systems that do not share similar structures and demographic profiles. Nonetheless, we have aimed to provide a comprehensive description of the development process using an implementation science approach, which could serve as a blueprint for other studies.

## 6 Conclusion

This study outlined a comprehensive approach to developing a transitional care model tailored to meet the complex needs of frail older adults. By leveraging principles of implementation science, we addressed specific challenges associated with the transition from hospital to home, enhancing the model's adaptability in diverse settings. Through iterative pilot testing and extensive engagement of project interest groups, we have shown that these methods are crucial for improving the contextual fit of an evidence‐based model. Looking forward, it will be important to focus on evaluating the effectiveness of specific components of the TCM to optimise their impact on patient outcomes. The logic model developed in this study offers a guiding framework for these evaluations, providing a structured approach to optimise patient outcomes.

## Author Contributions

C.H.N., O.Ma., O.Mu., C.O.G., I.P., T.B. made substantial contributions to conception and design, or acquisition of data, or analysis and interpretation of data. C.H.N., O.Ma., O.Mu., C.O.G., I.P., T.B. involved in drafting the manuscript or revising it critically for important intellectual content. C.H.N., O.Ma., O.Mu., C.O.G., I.P., T.B. gave final approval of the version to be published. Each author should have participated sufficiently in the work to take public responsibility for appropriate portions of the content. C.H.N., O.Ma., O.Mu., C.O.G., I.P., T.B. agreed to be accountable for all aspects of the work in ensuring that questions related to the accuracy or integrity of any part of the work are appropriately investigated and resolved.

## Conflicts of Interest

The authors declare no conflicts of interest.

## Peer Review

The peer review history for this article is available at https://www.webofscience.com/api/gateway/wos/peer‐review/10.1111/jan.16822.

## Supporting information


**Table S1.** (a, b) Sociodemographic data of interviewees, step 1 (context analysis).

**Table S2.** Definition of core elements and minimal requirements of the AdvantAGE intervention.

## Data Availability

Data sharing not applicable to this article as no datasets were generated or analysed during the current study.
