# Supplementary material for: A Multi‐Method Study to Develop and Pilot Test an Interprofessional Transitional Care Model for Frail Older Adults – AdvantAGE
Source: J Adv Nurs. 2025 Mar 12;81(11):7896–908. doi: 10.1111/jan.16822 (PMC12535312; doi:10.1111/jan.16822)
Supplement: Supplementary file 1 — Table S1. (a, b) Sociodemographic data of interviewees, step 1 (context analysis). [file JAN-81-7896-s002.docx]

Supportive Information: Table S1 (S1a and S1b): Characteristics of Interviewees

Table S1a: Characteristics of patients and caregivers (group A)

|  | Patients, n= 4 | Caregiver, n=3 |
| --- | --- | --- |
| Female | 2 | 2 |
| **Age, years** | | |
| 70 and younger | 1 | 2 |
| 71-80 | 0 | 0 |
| 81-90 | 1 | 1 |
| 91 and older | 2 |  |
| **Living Situation** | | |
| living alone | 4 |  |
| **Relationship** | | |
| spouse |  | 1 |
| child |  | 1 |
| other |  | 1 (niece) |

Table S1b: Characteristics of healthcare providers (group B and C)

|  | Health care staff, acute setting, n= 19 | Health care staff, community setting, n=18 |
| --- | --- | --- |
| Female | 9 | 9 |
| **Age, years** | | |
| 30 and younger | 3 | 2 |
| 31-39 | 4 | 4 |
| 40-49 | 4 | 4 |
| 50 and older | 8 | 8 |
| **Profession** | | |
| Registered Nurse | 9 |  |
| Physiotherapist/ occupational therapist | 5 | 1 |
| APN |  | 2 |
| Physician (hospital) | 4 |  |
| GP |  | 7 |
| Social worker |  | 3 |
| Pharmacist | 1 | 3 |
| Homecare organisation |  | 1 |
| Social Community Counsellor |  | 1 |
| **Work experience** | | |
| less than 5 years | 2 | 1 |
| 5-10 years | 5 | 4 |
| 11-20 years | 2 | 7 |
| 21-30 years | 5 | 6 |
| more than 30 years | 5 | 0 |
