# Supplementary material for: A Multi‐Method Study to Develop and Pilot Test an Interprofessional Transitional Care Model for Frail Older Adults – AdvantAGE
Source: J Adv Nurs. 2025 Mar 12;81(11):7896–908. doi: 10.1111/jan.16822 (PMC12535312; doi:10.1111/jan.16822)
Supplement: Supplementary file 2 — Table S2. Definition of core elements and minimal requirements of the AdvantAGE intervention. [file JAN-81-7896-s001.docx]

Table S2: Definition of core elements and minimal requirements of the AdvantAGE intervention

| **Intervention elements** | **Minimal Requirements** |
| --- | --- |
| **(1) Continuous support for patients and caregivers** |  |
| 1. Develop a treatment plan based on the individual goals of patients | - Patient care goals are clarified and documented after the initial home visit. - The AdvantAGE procedure is recorded in the patient's record. |
| 1. Primary point of contact for patients and their families in managing illness and addressing care-related issues | - A document listing emergency contact numbers is provided to the patient during the first home visit. - The patient's caregiver is contacted within the first week after hospital discharge unless the patient objects to this contact. |
| 1. Relationship building with patients | - Over 70% of home visits are conducted by the same Advanced Practice Nurse |
| **(2) Care coordination with primary care providers** |  |
| 1. Communicate relevant information to all parties for knowledge exchange | - An appointment with the patient’s General Practitioner (GP) is scheduled before the patient is discharged. - The patient’s current medication list, the hospital discharge letter, any existing advance directives, and the GP's telephone number are placed in the patient's home where they are easily accessible to other caregivers in emergencies |
| 1. Coordinate with GPs for additional testing and measures | - The responsible GP is informed of the patient’s participation in the AdvantAGE program and their current condition after the initial home visit. - The responsible GP and home care team receive a final report at least two weeks after the AdvantAGE program concludes. |
| **(3) Comprehensive health management at home** |  |
| 1. Monitor health status | - Before discharge, the AdvantAGE APN and physician determine the focus of clinical monitoring and the treatment goal for the program. - Diagnosis-specific clinical assessments and vital sign checks are performed at each visit or at least weekly, with results documented. - During the first four weeks, at least one home visit is conducted weekly |
| 1. Evaluate the effectiveness of treatments initiated during hospitalization | - All interventions recommended in the discharge letter are coordinated within one week of discharge. - Patient therapy adherence is assessed and documented after each home visit. |
| 1. Assessment of living situations | - A comprehensive case history is taken during the first home visit |
| **(4) Medication- and self- management with patients and caregivers** |  |
| 1. Reconcile and adjust medications and educate patients | - Availability of a current medication list is ensured. - Availability of prescribed medications is secured. - During the first home visit, patients are asked to display all medications they have at home. - Patients are interviewed about their medication routines, including what medications they take, how they take them, and at what times. - The medications present are compared to those on the current medication list. - Any discrepancies between prescribed medications and actual intake are documented and addressed. - A consultation on medication management is held within the first two weeks after discharge. |
| 1. Promote and support self-management for chronic conditions | - Patients are provided with information on individually relevant symptom management before discharge. - Instruction on symptoms and symptom management is given by the second home visit. - At least once during the AdvantAGE program, a detailed consultation is conducted focusing on managing one or more chronic conditions. |
| **(5) Advanced Care Planning** |  |
| 1. Advise and discuss on advanced care planning | - Advanced Care Planning is offered during the program   - If declined, the rejection and any documented patient wishes are added to the patient’s history.   - If the patient consents, an advanced directive is completed and forwarded to the GP and/or caregivers. |
